# Supplementary material for: Ferritinophagy mediates adaptive resistance to EGFR tyrosine kinase inhibitors in non-small cell lung cancer
Source: Nat Commun. 2024 May 17;15:4195. doi: 10.1038/s41467-024-48433-8 (PMC11101634; doi:10.1038/s41467-024-48433-8)
Supplement: Supplementary file 1 — Supplementary Information File [file 41467_2024_48433_MOESM1_ESM.pdf]

Ferritinophagy mediates adaptive resistance to EGFR tyrosine kinase inhibitors in non-small cell lung cancer

Supplementary Figures and figure legends 1-10

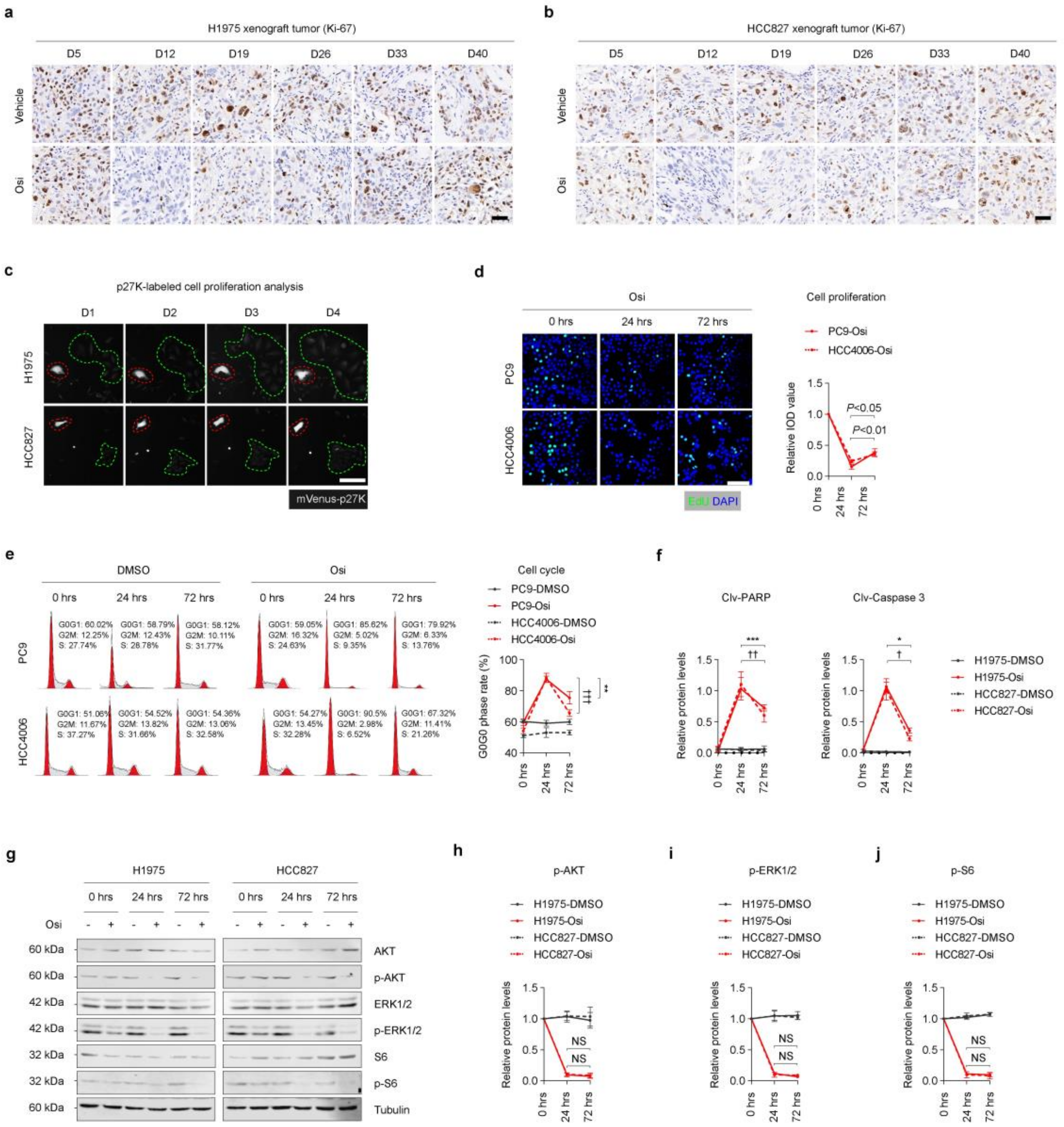

Supplementary Fig. 1 Osi induced adaptive resistance was not due to downstream EGFR reactivation.

a, b BALB/c nude mouse subcutaneously inoculated with H1975 or HCC827 cells were given Vehicle or Osi treatment. A subset of mice from each group were sacrificed, and their subcutaneous tumors were excised for IHC analysis (n = 9 samples each group). Images displaying the levels of tumor Ki-67 expression were taken on days 5 (the final day prior to treatment), 12, 26, 33, and 40 (the conclusion of treatment) for each group of mice. Scale bar, 50 μm**. c** Representative images of H1975 and HCC827 stably transfected with mVenus-p27K in day 1, day 2, day 3 and day 4. The red dotted line shows cells in a resting state, and the green dotted line shows cells in a proliferative state (n = 4 technical replicates; 3 independent experiments). Scale bar, 50 μm. **d** Left, Representative images of PC9

(EGFR Exon 19_DEL) and HCC4006 (EGFR Exon 19_DEL) cells treated with Osi for 0 hours, 24 hours, or 72 hours and incubated with 5-ethynyl-2'-deoxyuridine (EdU) for 2 hours. Scale bar, 100 μm. Right, quantitative of the relative intensity of EdU. Representative experiment (n = 3 technical replicates; 3 independent experiments). **e** Left, flow cytometry analysis of cell cycles of PC9 and HCC4006 cells treated with DMSO or Osi for 0 hours, 24 hours, or 72 hours. Right, percentage of G0 & G1 phases of PC9 (p=0.003) and HCC4006 (p<0.001) cells treated with DMSO or Osi for 0 hours, 24 hours or 72 hours. Representative experiment (n = 3 technical replicates; 3 independent experiments). **f** Immunoblotting analysis of apoptosis-related proteins poly ADP-ribose polymerase (PARP) and Capase-3 in H1975 and HCC827 cells treated with DMSO or Osi for 0 hours, 24 hours or 72 hours (n = 3 independent experiments). β-Actin (ACTB) is internal control. The figure presents a quantitative analysis of cleaved PARP and Caspase 3. **g-j** Immunoblotting analysis of EGFR downstream phosphorylated AKT (p-AKT), phosphorylated ERK1/2 (pERK1/2) and phosphorylated S6 (p-S6) in H1975 and HCC827 cells treated with DMSO or Osi for 0 hours, 24 hours or 72 hours (n = 3 technical replicates; 1independent experiment). Tubulin is internal control. Data are shown as mean ± SD and were analyzed by a two-way ANOVA (**d-j**). NS = no significance, **p < 0.01. $^{\dagger}p<0.05$, $^{\dagger\dagger\dagger}p < 0.001$, which was employed to specifically demonstrate the distinction in HCC827 cells. Source data are provided as a Source Data file.

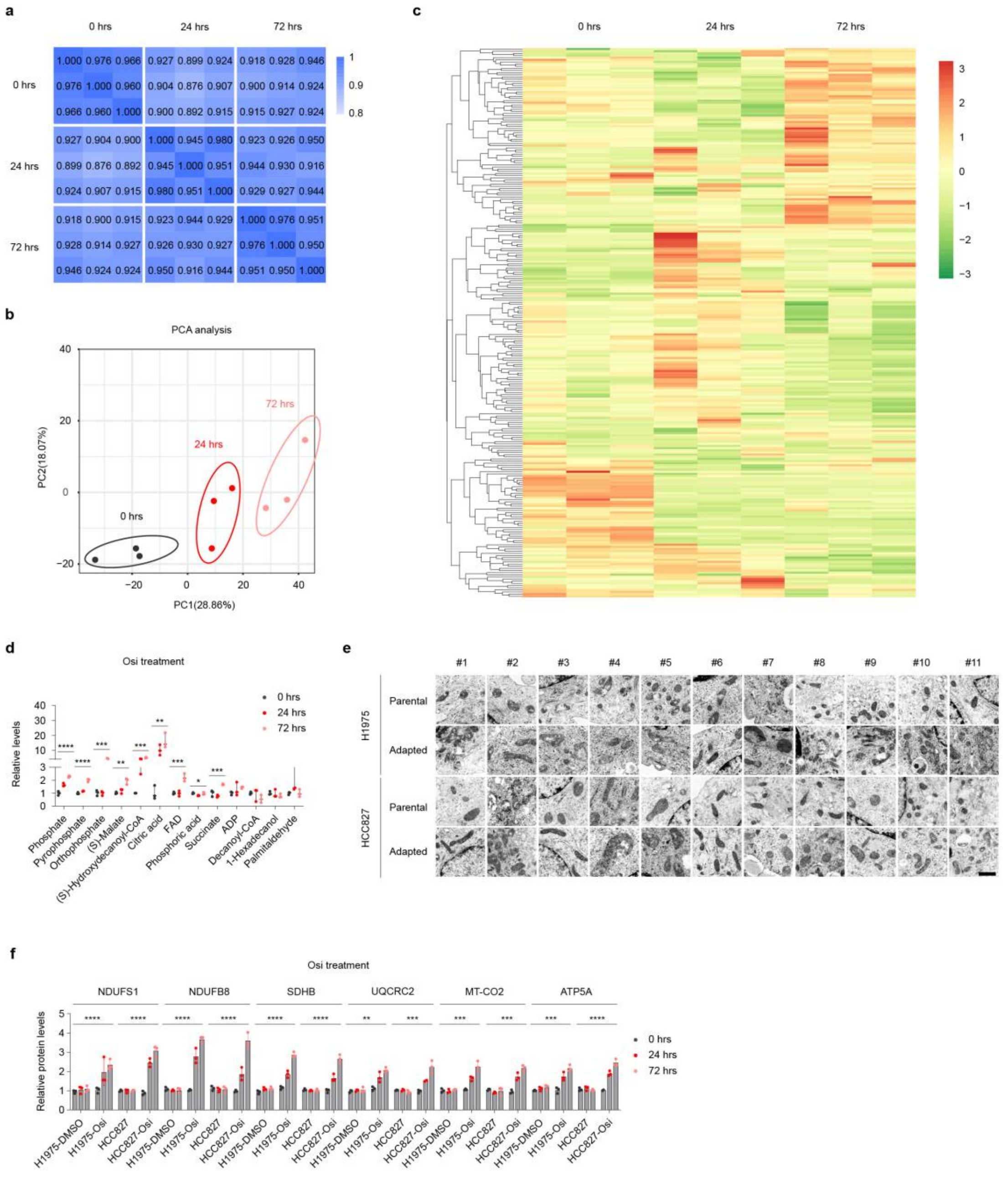

Supplementary Fig. 2 Metabolomics sequencing analyses metabolic characteristics in adaptive-resistant cells.

a Heat maps show differences of metabolites within each group in H1975 cells treated with Osi for 0 hours, 24 hours, or 72 hours (n = 3 samples). **b** Principal component analysis (PCA) of metabolites in H1975 cells treated with Osi for 0 hours, 24 hours, or 72 hours (n = 3 samples). **c** Heat maps displaying different metabolites after 24 hours or 72 hours Osi treatment in H1975 cells (n = 3 samples). **d** Quantitative analysis of metabolites associated with OXPHOS from metabolite sequencing data (n = 3 samples). **e** H1975 and HCC827 cells were harvested after 0 hours (Parental) or 72 hours (Adapted) Osi treatment and analyzed by electron microscopy for mitochondrial morphology. Representative experiment (n =12 technical replicates; 3 independent experiments). Scale bar, 1 μm. **f**

Representative immunoblotting of electron transport chain (ETC) proteins in H1975 and HCC827 cells treated with DMSO or Osi for 0 hours, 24 hours, and 72 hours (n = 3 independent experiments). β-Actin (ACTB) is internal control. Data are shown as mean ± SD and were analyzed by a one-way ANOVA (**d**) or a two-way ANOVA (**f**). NS = no significance, **p < 0.01, ***p < 0.001, ***p<0.0001. Source data are provided as a Source Data file.

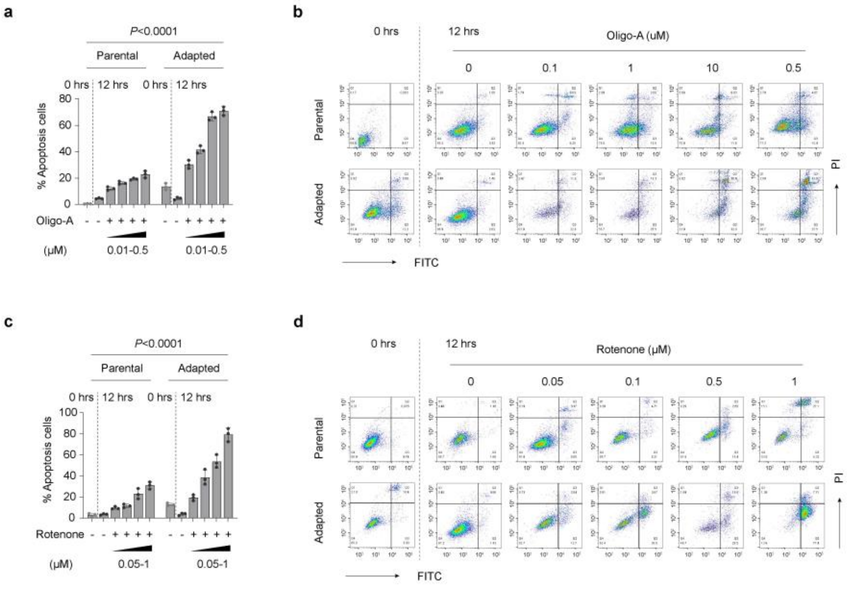

Supplementary Fig. 3 Osi adapted cells are dependent on OXPHOS.

a-d Apoptosis levels of parental and adapted H1975 cells at 0 hours, treated with DMSO for 12 hours, and treated with an increasing concentration of oligo-A (0.01-0.5 μM) or rotenone (0.05-1 μM) for 12 hours. Representative experiment (n = 3 technical replicates; 3 independent experiments). Data are shown as mean ± SD and were analyzed by a two-way ANOVA. Source data are provided as a Source Data file.

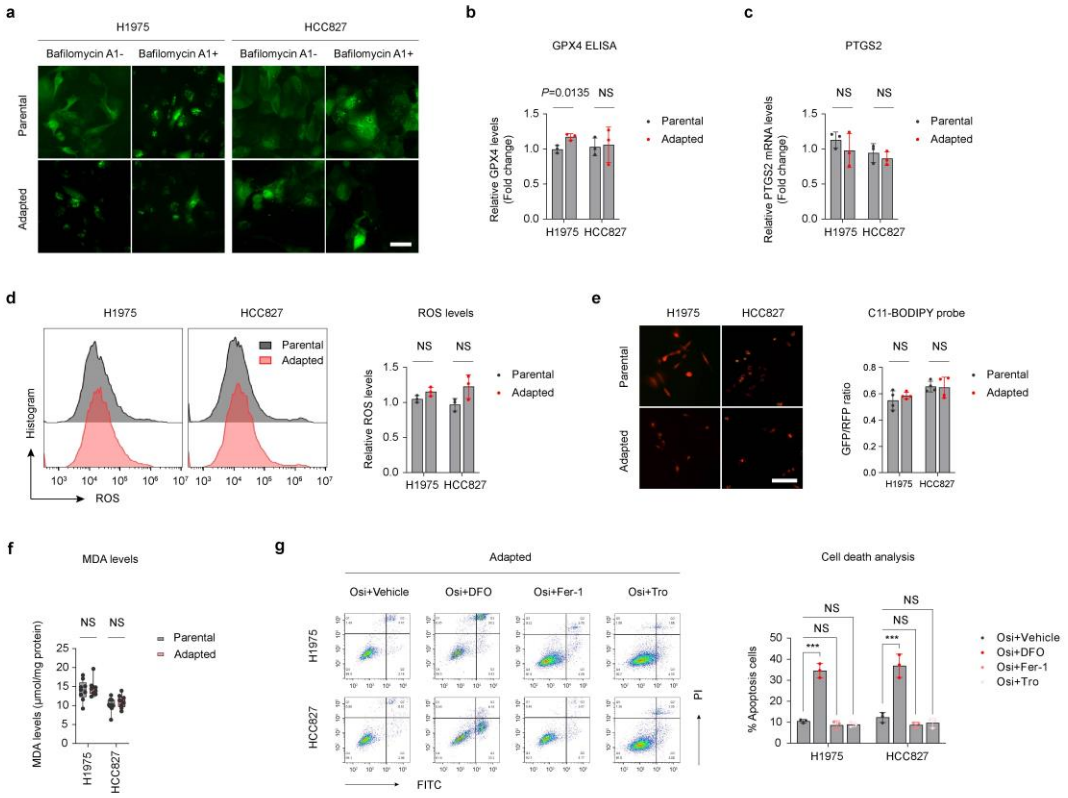

Supplementary Fig. 4 Activated ferritinophagy in adaptive resistance does not lead to Ferroptosis.

a Representative image of H1975 and HCC827 cells stably transfected with mCherry-EGFP-LC3B under the treatments of Osi for 0 hours (Parental) or 72 hours (Adapted) followed by two hours of treatment with DMSO or Bafilomycin A1 (n = 3 technical replicates; 3 independent experiments). Scale bar, 20 μm. **b** The expression of GPX4 in H1975 and HCC827 cells was detected by Enzyme-Linked Immunosorbent Assay (ELISA) after 0 hours (Parental) or 72 hours (Adapted) Osi treatment. Representative experiment (n = 3 technical replicates; 3 independent experiments). **c** PTGS2 mRNA expression levels was measured by qRT-PCR in H1975 and HCC827 cells treated with DMSO or Osi for 0 hours (Parental) or 72 hours (Adapted). Representative experiment (n = 3 technical replicates; 3 independent experiments). **d** H1975 and HCC827 cells were collected after 0 hours (parental) or 72 hours (adapted) Osi treatment and incubated with DCFH-DA for 20 minutes, and Reactive oxygen species (ROS) levels was analyzed by flow cytometry analysis. Representative experiment (n = 3 technical replicates; 3 independent experiments). **e** Representative image of H1975 and HCC827 cells treated with Osi for 0 hours (Parental) or 72 hours (Adapted) followed by 2 hours of C11-BODIPY probe staining (n = 4 technical replicates; 3 independent experiments). Scale bar, 50 μm. **f** Lipid Peroxidation (MDA) levels of H1975 and HCC827 cells was detected after 0 hours (Parental) or 72 hours (Adapted) Osi treatment. Representative experiment (n = 9 technical replicates; 3 independent experiments). **g** Flow cytometry analysis analysis of cell apoptosis in adapted H1975 and HCC827 cells after 12 hours of Osi, Osi combined with Deferoxamine (DFO), Osi combined with Ferrostatin-1 (Fer-1), or Osi combined with Trolox (TRO) treatment. Representative experiment (n = 3 technical replicates; 3 independent experiments). Data

are shown as mean ± SD and were analyzed by a two-tailed unpaired t-test (**b-f**) or a one-way ANOVA (**g**). NS = no significance, **p < 0.01, ***p < 0.001, ***p<0.0001. Source data are provided as a Source Data file.

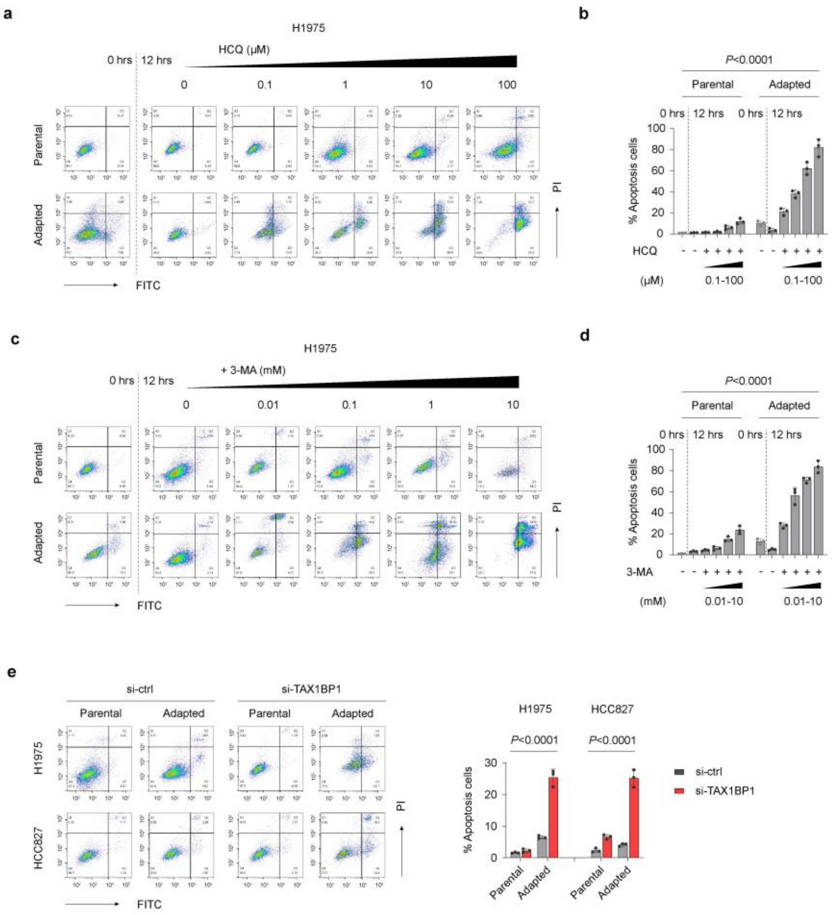

Supplementary Fig. 5 Osi adapted cells are sensitive to inhibition of ferritinophagy. **a-d** Apoptosis levels of parental and adapted H1975 cells at 0 hours, treated with DMSO for 12 hours, and treated with an increasing concentration of hydroxychloroquine (HCQ, 0.1-100 μM) or 3-Methyladenine (3-MA, 0.01-10 mM) for 12 hours. Representative experiment (n = 3 technical replicates; 3 independent experiments). **e** Apoptosis levels of H1975 or HCC827 cells transfected with si-ctrl or si-TAX1BP1 for 24 hours followed by 0 hours (Parental) or 72 hours Osi (Adapted) treatment. Representative experiment (n = 3 technical replicates; 3 independent experiments). Data are shown as mean ± SD and were analyzed by a two-way ANOVA. Source data are provided as a Source Data file.

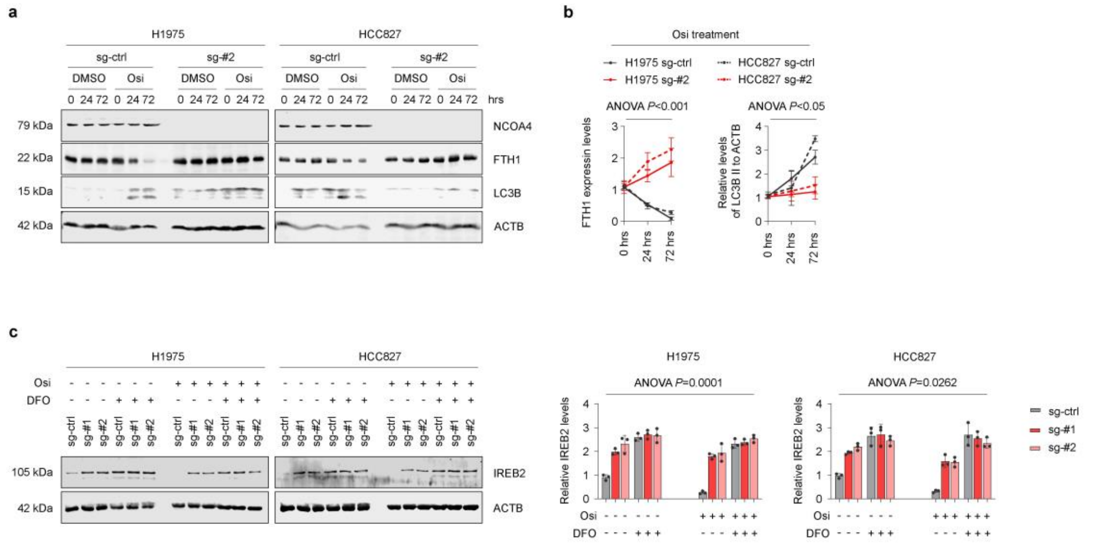

Supplementary Fig. 6 Knockout of NCOA4 inhibits the ferritinophagy of Osi adapted cells.

a, b Immunoblotting analysis of cell lysates was performed to determine the levels of NCOA4, FTH1 and LC3B in NCOA4 sg-ctrl or sg-#2 H1975 and HCC827 cells treated with Osi for 0 hours, 24 hours or 72 hours (Representative images from 3 independent experiments). ACTB is internal control. **c** The expression levels of IREB2 were assessed using immunoblotting analysis in NCOA4 sg-ctrl, sg-#1, sg-#2 H1975, and HCC827 cells after a 72-hour treatment with DMSO/Osi. The addition of the iron chelator Deferasirox (DFO) served as a positive control (n = 3 technical replicates; 1 independent experiment). ACTB is internal control. Data are shown as mean ± SD and were analyzed by a two-way ANOVA. Source data are provided as a Source Data file.

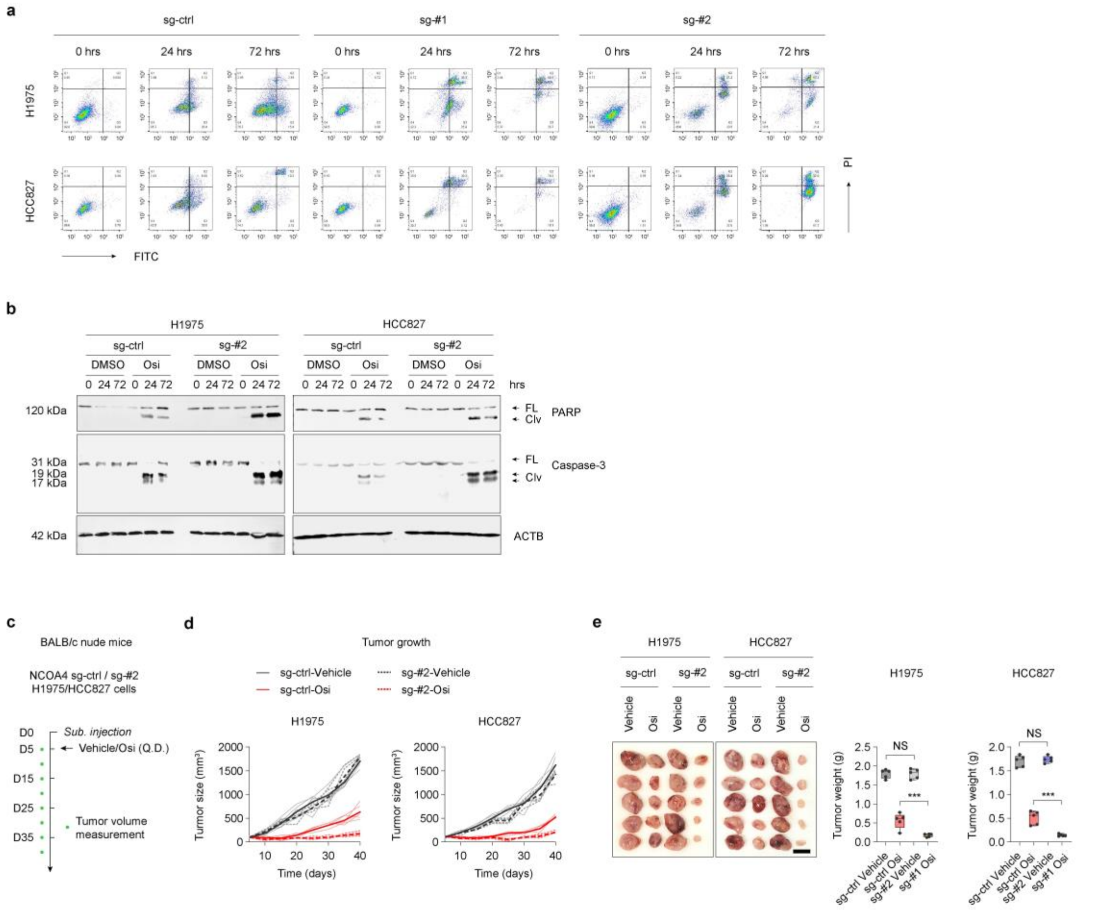

Supplementary Fig. 7 Knockout of NCOA4 inhibits the formation of Osi adapted resistance.

a Apoptosis levels of NCOA4 sg-ctrl, sg-#1 and sg-#2 H1975 or HCC827 cells followed by 0 hours, 24 hours and 72 hours Osi treatment. Representative experiment (n = 3 technical replicates; 3 independent experiments). **b** Immunoblotting analysis of apoptosis-related proteins poly ADP-ribose polymerase (PARP) and Capase-3 (Cas-3) in NCOA4 sg-ctrl or sg-#2 H1975 and HCC827 cells treated with DMSO or Osi for 0 hours, 24 hours or 72 hours (n = 3 technical replicates; 1 independent experiment). β-Actin (ACTB) is internal control. **c, d** BALB/c nude mice were subcutaneous inoculated with NCOA4 sg-ctrl or sg-#2 H1975 and HCC827 cells and were given Vehicle (DMSO + saline) or Osi (nasal feeding, once a day) and follow-up (n = 5 mice each group). **e** Tumor weight was measured for Vehicle-treated group and Osi-treated group (n = 5 mcie each group). Scale bar, 1 cm. Data are shown as mean ± SD and were analyzed by a one-way ANOVA. Source data are provided as a Source Data file.

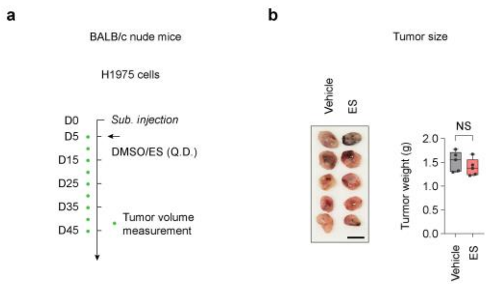

Supplementary Fig. 8 Elesclomol alone does not inhibit tumor growth.

a BALB/c nude mice were subcutaneous inoculated with H1975 cells and were given Vehicle (DMSO + saline) or elesclomol (ES) and follow-up (n = 5 mcie each group). **b** Tumor weight was measured for Vehicle-treated group and ES-treated (n = 5 mice each group). Scale bar, 1 cm. Data are shown as mean ± SD and were analyzed by a two-tailed unpaired t-test. NS = no significance. Source data are provided as a Source Data file.

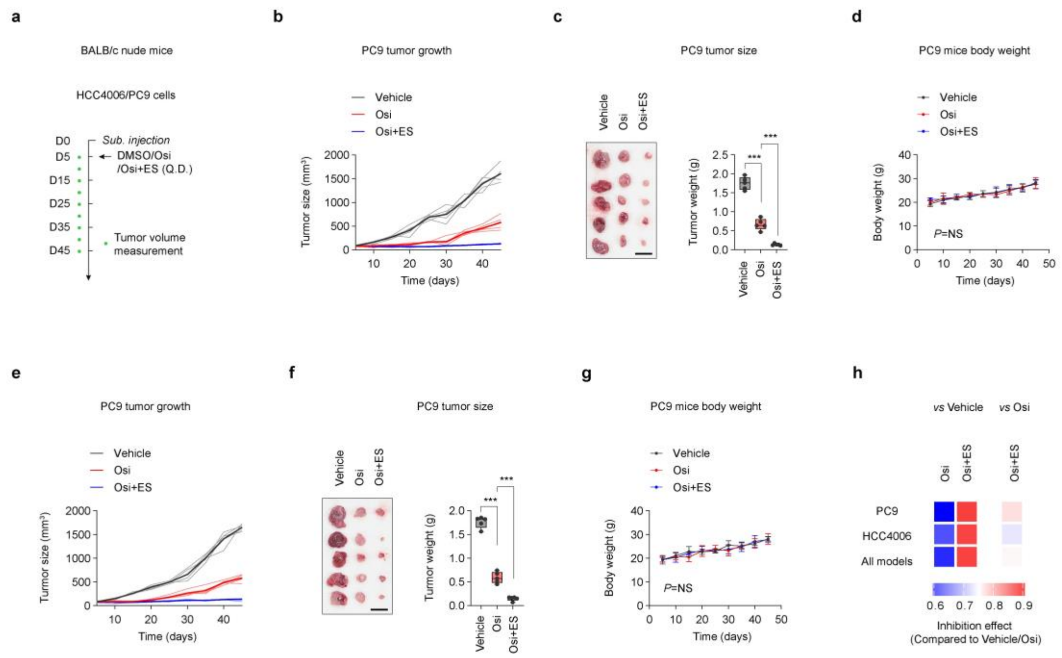

Supplementary Fig. 9 Active Fe-S protein synthesis in Osi adaptive resistant tumor cells significantly reduced the threshold of Cuproptosis.

a, b, e BALB/c nude mice were subcutaneous inoculated with PC9 or HCC4006 cells and were given Vehicle (DMSO + saline), Osi (nasal feeding, once a day), or Osi plus elesclomol (Osi + ES) and follow-up (n = 5 mice each group). **c, f** Tumor weight was measured for Vehicle-treated group, Osi-treated group and Osi plus elesclomol-treated (Osi + ES) group (n = 5 mice each group). Scale bar, 1 cm. **d, g** Mouse weight was measured from **f** as a surrogate for treatment toxicity (n = 5 mice each group). **h** Generalized linear models to test the association of change in tumor volume over time, either between Osi treatment groups and vehicle (left) or between combination therapy and Osi alone (right) (n = 5 mice each group). Data are shown as mean ± SD and were analyzed by a one-way ANOVA (**c, f**) or a two-way ANOVA (**d, g**). NS = no significance, ***p < 0.001. Source data are provided as a Source Data file.

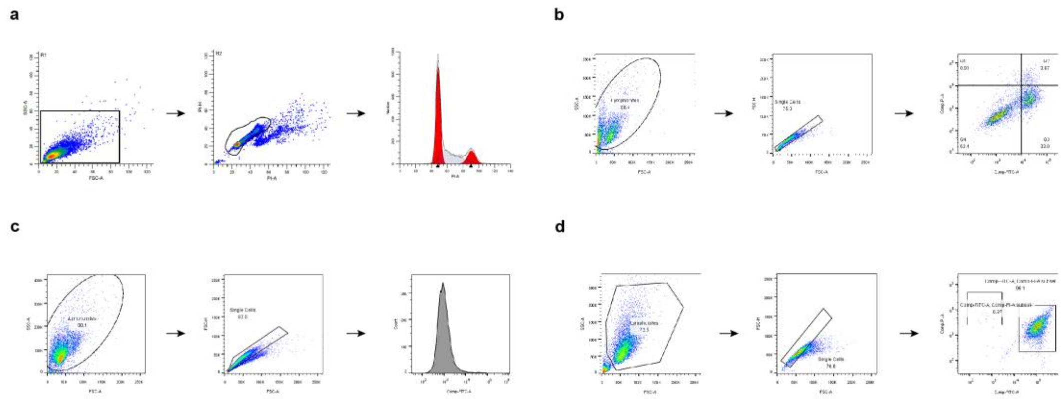

Supplementary Fig. 10 Gating strategy of flow cytometry analysis.

a Gating strategy of cell cycle analysis in Fig. 1k and Supplementary Fig. 1e. **b** Gating strategy of PI/Annexin V apoptosis assay in Fig. 1l, Fig. 6c, Supplementary Fig. 3a-d, Supplementary Fig. 4g, Supplementary Fig. 5a-e and Supplementary Fig. 7a. **c** Gating strategy of cellular ROS analysis in Supplementary Fig. 4d. **d** Gating strategy of each cell's proportion analysis in Fig. 6i.

Supplementary Table 1 Compounds used in this study.

Reagent or resource	Source	Identifier	Concentration
DMSO	Solarbio	Cat# D9371	\
Osimertinib	MedChemExpress	Cat# HY-15772	500 nM / 10 mg/kg
Bafilomycin A1	MedChemExpress	Cat# HY-100558	200 nM
3-Methyladenine	MedChemExpress	Cat# HY-19312	5 μM
Hydroxychloroquine	MedChemExpress	Cat# HY-B1370A	10 μM / 100 mg/kg
D-luciferin potassium salt	Invitrogen	Cat# L2916	150 mg/kg
Oligomycin-A	Agilent	Cat# 103673-100	1 μM
FCCP	Agilent	Cat# 103673-100	0.5 μM
Antimycin A	Agilent	Cat# 103673-100	0.5 μM
Rotenone	Agilent	Cat# 103673-100	0.5 μM
Elesclomsl	MedChemExpress	Cat# HY-12040	0.1 – 100 nM
Disulfiram	MedChemExpress	Cat# HY-B0240	0.1 – 100 nM
NSC-319726	MedChemExpress	Cat# HY-18634	0.1 – 100 nM
$CuCl_2$	MilliporeSigma	Cat# 231-210-2	0.1 – 100 nM

Supplementary Table 2 The clinical characteristics of the patients

No	EGFR mutation	PFS	status	sex	age	stage	histology	differentiation	smoking	ECOG PS	liver M	bone M	brain M	response	NCOA4 expression
1	19_DEL	43.17	1	F	70	IVA	LUAD	Low	Never	1	Yes	No	No	Yes	Low
2	21_L858R	34.43	1	F	63	IVB	LUAD	Low	Never	1	No	No	Yes	Yes	Low
3	19_DEL	32.87	0	M	70	IVA	LUAD	Low	Current/Former	1	No	No	Yes	No	Low
4	19_DEL	29.53	0	M	55	IVB	LUAD	Low	Current/Former	1	Yes	No	No	No	Low
5	19_DEL	28	1	M	52	IVA	LUAD	Low	Current/Former	2	No	No	Yes	Yes	Low
6	19_DEL	27.67	1	M	81	IVA	LUAD	Low	Never	1	No	Yes	No	Yes	Low
7	21_L858R	27.1	1	M	70	IVB	LUAD	Low	Never	1	No	Yes	Yes	Yes	Low
8	19_DEL	26.63	0	M	46	IVA	LUAD	Low	Current/Former	0	No	Yes	No	Yes	High
9	19_DEL	25.43	0	M	59	IVA	LUAD	Low	Never	1	No	Yes	No	Yes	Low
10	19_DEL	19.8	1	M	62	IVA	LUAD	Medium/High	Never	1	No	No	Yes	Yes	High
11	21_L858R	19.77	0	M	53	IVA	LUAD	Low	Never	1	No	Yes	No	Yes	Low
12	21_L858R	18.97	0	M	51	IIIC	LUAD	Low	Current/Former	1	No	No	No	No	High
13	19_DEL	18.8	1	F	79	IVB	LSCC	Low	Never	2	Yes	No	No	Yes	High
14	19_DEL	18.5	0	M	65	IVA	LUAD	Low	Current/Former	1	No	Yes	No	Yes	Low
15	19_DEL	18.23	1	M	57	IVA	LUAD	Medium/High	Never	1	Yes	No	No	Yes	High
16	19_DEL	18.1	0	F	70	IVA	LUAD	Low	Never	1	No	Yes	No	Yes	Low
17	21_L858R	17.63	1	M	53	IVB	LUAD	Low	Current/Former	1	No	Yes	Yes	Yes	Low
18	19_DEL	17.33	1	M	65	IVA	LUAD	Low	Never	0	No	Yes	No	Yes	Low
19	19_DEL	16.93	1	M	68	IVA	LUAD	Low	Current/Former	1	Yes	No	No	Yes	Low
20	21_L858R	16	1	M	74	IVA	LSCC	Low	Never	1	No	Yes	No	Yes	High
21	19_DEL	15.67	1	F	42	IVA	LUAD	Medium/High	Never	1	No	Yes	No	Yes	Low

22	21_L858R	15.1	1	M	70	IVA	LUAD	Low	Current/Former	1	No	No	Yes	Yes	Low
23	21_L858R	14.17	1	M	53	IVA	LUAD	Low	Never	1	No	Yes	No	Yes	High
24	19_DEL	13.83	1	M	77	IVA	LSCC	Medium/High	Current/Former	0	Yes	No	No	Yes	Low
25	19_DEL	13.27	1	F	45	IVB	LUAD	Low	Never	1	Yes	No	No	No	High
26	21_L858R	13.17	0	M	55	IVA	LUAD	Medium/High	Never	1	No	Yes	No	Yes	High
27	19_DEL	11.73	1	F	73	IVA	LUAD	Low	Never	1	No	Yes	No	Yes	High
28	19_DEL	11.47	0	M	67	IVB	LUAD	Low	Never	1	No	Yes	Yes	No	High
29	21_L858R	10.97	1	F	50	IVA	LUAD	Medium/High	Never	1	Yes	No	No	Yes	High
30	19_DEL	10.63	1	M	51	IVA	LUAD	Low	Current/Former	1	Yes	No	No	Yes	Low
31	21_L858R	10.5	1	M	67	IVB	LUAD	Low	Never	1	No	No	Yes	Yes	Low
32	19_DEL	10.33	1	M	48	IVA	LUAD	Low	Current/Former	0	No	Yes	No	No	High
33	21_L858R	10.13	0	F	48	IVB	LUAD	Low	Never	1	No	No	Yes	No	High
34	21_L858R	10.07	1	M	74	IVB	LSCC	Low	Current/Former	1	Yes	No	No	Yes	High
35	21_L858R	9.8	1	M	60	IVB	LSCC	Low	Never	1	Yes	No	Yes	No	High
36	19_DEL	9.23	1	F	69	IVB	LUAD	Low	Never	1	Yes	No	Yes	No	Low
37	21_L858R	7.57	1	F	77	IVA	LUAD	Low	Never	1	Yes	No	No	No	High
38	19_DEL	5.77	1	M	72	IVA	LUAD	Low	Current/Former	1	No	No	Yes	No	High
39	21_L858R	4	1	M	49	IVB	LUAD	Low	Current/Former	1	No	No	Yes	No	High
40	21_L858R	3.97	1	M	70	IIIC	LUAD	Low	Never	1	No	Yes	No	No	High

**Supplementary Table 3 The sequences of primers used in this study.**		
**Primers for PCR (5’-3’)**		**concentration**
NCOA4-F	GAGGTGTAGTGATGCACGGAG	1μmol
NCOA4-R	GACGGCTTATGCAACTGTGAA	1μmol
PTGS2-F	CTGGCGCTCAGCCATACAG	1μmol
PTGS2-R	GCCTTGGAGATGAGCAGGAT	1μmol
ACTB-F	CATGTACGTTGCTATCCAGGC	1μmol
ACTB-R	CTCCTTAATGTCACGCACGAT	1μmol
