# Supplementary material for: Ferritinophagy mediates adaptive resistance to EGFR tyrosine kinase inhibitors in non-small cell lung cancer
Source: Nat Commun. 2024 May 17;15:4195. doi: 10.1038/s41467-024-48433-8 (PMC11101634; doi:10.1038/s41467-024-48433-8)
Supplement: Supplementary file 5 — Reporting Summary [file 41467_2024_48433_MOESM5_ESM.pdf]

nature portfolio

Corresponding author(s): Feng Jiang, Gaochao Dong

Last updated by author(s): Feb 25, 2024

Reporting Summary

Nature Portfolio wishes to improve the reproducibility of the work that we publish. This form provides structure for consistency and transparency in reporting. For further information on Nature Portfolio policies, see our Editorial Policies and the Editorial Policy Checklist.

Statistics

For all statistical analyses, confirm that the following items are present in the figure legend, table legend, main text, or Methods section.

n/a	Confirmed	
☐	☒	The exact sample size (*n*) for each experimental group/condition, given as a discrete number and unit of measurement
☐	☒	A statement on whether measurements were taken from distinct samples or whether the same sample was measured repeatedly
☐	☒	The statistical test(s) used AND whether they are one- or two-sided<br>*Only common tests should be described solely by name; describe more complex techniques in the Methods section.*
☒	☐	A description of all covariates tested
☐	☒	A description of any assumptions or corrections, such as tests of normality and adjustment for multiple comparisons
☐	☒	A full description of the statistical parameters including central tendency (e.g. means) or other basic estimates (e.g. regression coefficient) AND variation (e.g. standard deviation) or associated estimates of uncertainty (e.g. confidence intervals)
☐	☒	For null hypothesis testing, the test statistic (e.g. *F*, *t*, *r*) with confidence intervals, effect sizes, degrees of freedom and *P* value noted<br>*Give P values as exact values whenever suitable.*
☒	☐	For Bayesian analysis, information on the choice of priors and Markov chain Monte Carlo settings
☐	☒	For hierarchical and complex designs, identification of the appropriate level for tests and full reporting of outcomes
☒	☐	Estimates of effect sizes (e.g. Cohen's *d*, Pearson's *r*), indicating how they were calculated

Our web collection on statistics for biologists contains articles on many of the points above.

Software and code

Policy information about availability of computer code

Data collection	QuantStudio 6 Flex system (Applied Biosystems) and ViiA 7 Dx system (Applied Biosystems) for qPCR; LSM 880 (Zeiss) confocal microscope for IF and FISH; Nanodrop 2000 spectrophotometer (Thermo) for absorbance; ODYSSEY CLx (LICOR) for IB; Glomax Luminometer (Promega) for Luciferase activity; IVIS Spectrum (PerkinElmer) for bioluminescence imaging analysis; LSRII flow cytometer (BD Biosciences) for FACS; QE-HFX mass spectrometer (Thermo) for MS; JEM-1010 (JEOL) for electron microscope photograph.
Data analysis	FlowJo software (version 10.0) for FACS analysis; Image Studio (5.x CLx) for IB analysis; QuantStudio Real-Time PCR Software (version 1.3) for PCR analysis; ZEN (version 3.1) for FISH and IF.<br>All data were obtained from independent experiments with independent biological replicates. The sample size was not predetermined using statistical methods. Most results were qualitatively replicated in two different cell lines and provided consistent results using independent techniques. No experiment or sample was excluded during the experiment and outcome assessment. Experimental data are presented as mean and individual points, mean and standard deviation as indicated in the figure legend. Immunofluorescence experiments were performed in at least three independent biological replicates. Before statistical analysis, tests were performed with the assumption that values followed a normal distribution and had similar variances. To assess the differences between a control group and an experimental group, an unpaired two-tailed Student's t-test was employed. One-way or multivariate ANOVA was utilized to test for the statistical significance of a control group and multiple experimental groups, and the Dunnett-t test was employed to evaluate pairwise differences between means of multiple samples in experimental tests. For certain specialized statistical methods, the corresponding details can be found in the figure legends that correspond to the results. SPSS v.28.0 and Prism v.9.4 were used for statistical analysis.

For manuscripts utilizing custom algorithms or software that are central to the research but not yet described in published literature, software must be made available to editors and reviewers. We strongly encourage code deposition in a community repository (e.g. GitHub). See the Nature Portfolio guidelines for submitting code & software for further information.

Data

Policy information about availability of data

All manuscripts must include a data availability statement. This statement should provide the following information, where applicable:

- Accession codes, unique identifiers, or web links for publicly available datasets
- A description of any restrictions on data availability
- For clinical datasets or third party data, please ensure that the statement adheres to our policy

The metabolomics data have been uploaded to the MetaboLights database, which is managed by the European Bioinformatics Institute (EBI) of EMBL (https://www.ebi.ac.uk/metabolights/). The dataset has been assigned the identifier MTBLS8108.
The RNA-seq data has been uploaded to the Gene Expression Omnibus (GEO) database (https://www.ncbi.nlm.nih.gov/geo/) and is associated with the accession number GSE236238.
The EGFR-wildtype and EGFR-mutant human NSCLC RNA expression data were derived from the TCGA Research Network: (https://portal.gdc.cancer.gov/projects/TCGA-LUAD; https://portal.gdc.cancer.gov/projects/TCGA-LUSC).
Source data are provided with this paper. All further data supporting the conclusions of this study are accessible upon reasonable request from the corresponding author. Correspondence and requests for materials should be addressed to Feng Jiang and Gaochao Dong.

Research involving human participants, their data, or biological material

Policy information about studies with human participants or human data. See also policy information about sex, gender (identity/presentation), and sexual orientation and race, ethnicity and racism.

Reporting on sex and gender

No sex
1 F
2 F
3 M
4 M
5 M
6 M
7 M
8 M
9 M
10 M
11 M
12 M
13 F
14 M
15 M
16 F
17 M
18 M
19 M
20 M
21 F
22 M
23 M
24 M
25 F
26 M
27 F
28 M
29 F
30 M
31 M
32 M
33 F
34 M
35 M
36 F
37 F
38 M
39 M
40 M

Reporting on race, ethnicity, or other socially relevant groupings

All patients in this study belong to the Asian population.

Population characteristics

No EGFR PFS status sex age stage histology diff smoking ECOG PS liverM boneM brainM response NCOA4 expression
1 19_DEL 43.17 1 F 70 IVA Lung Adenocarcinoma Low Never 1 Yes No No Yes Low
2 21_L858R 34.43 1 F 63 IVB Lung Adenocarcinoma Low Never 1 No No Yes Yes Low
3 19_DEL 32.87 0 M 70 IVA Lung Adenocarcinoma Low Current/Former 1 No No Yes No Low
4 19_DEL 29.53 0 M 55 IVB Lung Adenocarcinoma Low Current/Former 1 Yes No No No Low
5 19_DEL 28 1 M 52 IVA Lung Adenocarcinoma Low Current/Former 2 No No Yes Yes Low
6 19_DEL 27.67 1 M 81 IVA Lung Adenocarcinoma Low Never 1 No Yes No Yes Low
7 21_L858R 27.1 1 M 70 IVB Lung Adenocarcinoma Low Never 1 No Yes Yes Yes Low
8 19_DEL 26.63 0 M 46 IVA Lung Adenocarcinoma Low Current/Former 0 No Yes No Yes High
9 19_DEL 25.43 0 M 59 IVA Lung Adenocarcinoma Low Never 1 No Yes No Yes Low
10 19_DEL 19.8 1 M 62 IVA Lung Adenocarcinoma Medium/High Never 1 No No Yes Yes High
11 21_L858R 19.77 0 M 53 IVA Lung Adenocarcinoma Low Never 1 No Yes No Yes Low
12 21_L858R 18.97 0 M 51 IIIC Lung Adenocarcinoma Low Current/Former 1 No No No No High
13 19_DEL 18.8 1 F 79 IVB Lung Squamous Cell Carcinoma Low Never 2 Yes No No Yes High
14 19_DEL 18.5 0 M 65 IVA Lung Adenocarcinoma Low Current/Former 1 No Yes No Yes Low
15 19_DEL 18.23 1 M 57 IVA Lung Adenocarcinoma Medium/High Never 1 Yes No No Yes High
16 19_DEL 18.1 0 F 70 IVA Lung Adenocarcinoma Low Never 1 No Yes No Yes Low
17 21_L858R 17.63 1 M 53 IVB Lung Adenocarcinoma Low Current/Former 1 No Yes Yes Yes Low
18 19_DEL 17.33 1 M 65 IVA Lung Adenocarcinoma Low Never 0 No Yes No Yes Low
19 19_DEL 16.93 1 M 68 IVA Lung Adenocarcinoma Low Current/Former 1 Yes No No Yes Low
20 21_L858R 16 1 M 74 IVA Lung Squamous Cell Carcinoma Low Never 1 No Yes No Yes High
21 19_DEL 15.67 1 F 42 IVA Lung Adenocarcinoma Medium/High Never 1 No Yes No Yes Low
22 21_L858R 15.1 1 M 70 IVA Lung Adenocarcinoma Low Current/Former 1 No No Yes Yes Low
23 21_L858R 14.17 1 M 53 IVA Lung Adenocarcinoma Low Never 1 No Yes No Yes High
24 19_DEL 13.83 1 M 77 IVA Lung Squamous Cell Carcinoma Medium/High Current/Former 0 Yes No No Yes Low
25 19_DEL 13.27 1 F 45 IVB Lung Adenocarcinoma Low Never 1 Yes No No No High
26 21_L858R 13.17 0 M 55 IVA Lung Adenocarcinoma Medium/High Never 1 No Yes No Yes High
27 19_DEL 11.73 1 F 73 IVA Lung Adenocarcinoma Low Never 1 No Yes No Yes High
28 19_DEL 11.47 0 M 67 IVB Lung Adenocarcinoma Low Never 1 No Yes Yes No High
29 21_L858R 10.97 1 F 50 IVA Lung Adenocarcinoma Medium/High Never 1 Yes No No Yes High
30 19_DEL 10.63 1 M 51 IVA Lung Adenocarcinoma Low Current/Former 1 Yes No No Yes Low
31 21_L858R 10.5 1 M 67 IVB Lung Adenocarcinoma Low Never 1 No No Yes Yes Low
32 19_DEL 10.33 1 M 48 IVA Lung Adenocarcinoma Low Current/Former 0 No Yes No No High
33 21_L858R 10.13 0 F 48 IVB Lung Adenocarcinoma Low Never 1 No No Yes No High
34 21_L858R 10.07 1 M 74 IVB Lung Squamous Cell Carcinoma Low Current/Former 1 Yes No No Yes High
35 21_L858R 9.8 1 M 60 IVB Lung Squamous Cell Carcinoma Low Never 1 Yes No Yes No High
36 19_DEL 9.23 1 F 69 IVB Lung Adenocarcinoma Low Never 1 Yes No Yes No Low
37 21_L858R 7.57 1 F 77 IVA Lung Adenocarcinoma Low Never 1 Yes No No No High
38 19_DEL 5.77 1 M 72 IVA Lung Adenocarcinoma Low Current/Former 1 No No Yes No High
39 21_L858R 4 1 M 49 IVB Lung Adenocarcinoma Low Current/Former 1 No No Yes No High
40 21_L858R 3.97 1 M 70 IIIC Lung Adenocarcinoma Low Never 1 No Yes No No High

Recruitment

Human material was obtained from the Department of Thoracic Surgery, Jiangsu Cancer Hospital. Patients were not recruited specifically for this study.

Ethics oversight

Ethics permission was granted by the Jiangsu Cancer Hospital Medical Ethics Committee of Nanjing Medical University.

Note that full information on the approval of the study protocol must also be provided in the manuscript.

Field-specific reporting

Please select the one below that is the best fit for your research. If you are not sure, read the appropriate sections before making your selection.

[x] Life sciences [ ] Behavioural & social sciences [ ] Ecological, evolutionary & environmental sciences

For a reference copy of the document with all sections, see nature.com/documents/nr-reporting-summary-flat.pdf

Life sciences study design

All studies must disclose on these points even when the disclosure is negative.

Sample size

Sample size estimates has been performed on previous experience to obtain statistical significance and reproducibility. For in vivo studies, n=5-10 mice per group is sufficient to detect meaningful biological differences with good reproducibility. In each group, five mice were utilized in compliance with ethical standards, ensuring the reliability of the research findings while minimizing animal use as much as possible. For signaling assays, we typically performed at least three independent experiment repeats to allow statistical analysis and robust conclusions to be drawn. All sample sizes are listed in the corresponding figure legends or on the figures. All experiments were repeated at least three times.

Data exclusions	No data were excluded.
Replication	Replication All the findings were reliably reproduced in multiple independent experiments. For all experiments, our data represent at least two independent assays that produce similar results. We also used different assays and readouts to confirm our findings in different way.
Randomization	Animals used were randomly assigned to each treatment group. In experiments not involving animals, stratified randomization was predominantly utilized for cell experiments since many cell samples were subjected to an initial treatment before being randomly allocated to various subsequent treatments. Furthermore, confocal microscopy images were captured at random across all samples. The same number of cells were plated for each treatment condition and same amount of protein was used for co-IP and immunoblotting analyses.
Blinding	Following the random allocation of animals into groups, each group was subjected to different treatment protocols, which limited the feasibility of employing a blind method. Consequently, all animal studies were conducted using a non-blinded approach. Additionally, each experiment was independently carried out by two to three researchers, ensuring the repeatability of all results.

Reporting for specific materials, systems and methods

We require information from authors about some types of materials, experimental systems and methods used in many studies. Here, indicate whether each material, system or method listed is relevant to your study. If you are not sure if a list item applies to your research, read the appropriate section before selecting a response.

Materials & experimental systems

n/a	Involved in the study	
☐	☒	Antibodies
☐	☒	Eukaryotic cell lines
☒	☐	Palaeontology and archaeology
☐	☒	Animals and other organisms
☐	☒	Clinical data
☒	☐	Dual use research of concern
☒	☐	Plants

Methods

n/a	Involved in the study	
☒	☐	ChIP-seq
☐	☒	Flow cytometry
☒	☐	MRI-based neuroimaging

Antibodies

Antibodies used	The membranes were subjected to standard immunoblotting procedures using Alexa 680 and 800-conjugated species-specific secondary antibodies (Rockland). The membranes were visualized using an infrared scanner (LI-COR). The primary antibodies employed were as follows: anti-AKT (Cell Signaling, 9272S), anti-phospho-AKT Ser473 (Cell Signaling, 4060S), anti-ERK1/2 (Cell Signaling, 4695S), anti-phospho-ERK1/2 Thr202/Tyr204 (Cell Signaling, 4370S), anti-S6 (Cell Signaling, 2217S), anti-phospho-S6 Ser235/236 (Cell Signaling, 4858S), anti-PARP (Cell Signaling, 9542S), anti-Caspase-3 (Cell Signaling, 9662S), anti-NDUFS1 (Abcam, ab169540), anti-NDUFB8 (Abcam, ab192878), anti-SDHB (Abcam, ab175225), anti-UQCRC2 (Abcam, ab203832), anti-MTCO2 (Abcam, ab79393), anti-ATP5A (Abcam, ab14748), anti-NCOA4 (Cell Signaling, #66849), anti-FTH1 (Abcam, ab183781), anti-NDUFS3 (Abcam, ab177471), anti-UQCRFS1 (Abcam, ab191078), anti-FECH (Abcam, ab137042), anti-DPYD (Cell Signaling, #4654), anti-IREB2 (Abcam, ab181153), anti-DLAT (Cell Signaling, #12362), anti-DLST (Cell Signaling, # 5556), anti-Tubulin (Proteintech, 10068-1-AP) and anti-ACTB (Proteintech, 81115-1-RR). The antibodies were employed according to the manufacturer's recommended dilutions.
Validation	All antibodies used in our study have been validated and detailed information could be found on the website from manufactures. See following web sites for antibody validation by suppliers:<br>anti-AKT (Cell Signaling, 9272S)<br>https://www.cellsignal.cn/products/primary-antibodies/akt-antibody/9272<br>anti-phospho-AKT Ser473 (Cell Signaling, 4060S)<br>https://www.cellsignal.cn/products/primary-antibodies/phospho-akt-ser473-d9e-xp-174-rabbit-mab/4060<br>anti-ERK1/2 (Cell Signaling, 4695S)<br>https://www.cellsignal.cn/products/primary-antibodies/p44-42-mapk-erk1-2-137f5-rabbit-mab/4695<br>anti-phospho-ERK1/2 Thr202/Tyr204 (Cell Signaling, 4370S)<br>https://www.cellsignal.cn/products/primary-antibodies/phospho-p44-42-mapk-erk1-2-thr202-tyr204-d13-14-4e-xp-174-rabbit-mab/4370<br>anti-S6 (Cell Signaling, 2217S)<br>https://www.cellsignal.cn/products/primary-antibodies/s6-ribosomal-protein-5g10-rabbit-mab/2217<br>anti-phospho-S6 Ser235/236 (Cell Signaling, 4858S)<br>https://www.cellsignal.cn/products/primary-antibodies/phospho-s6-ribosomal-protein-ser235-236-d57-2-2e-xp-174-rabbit-mab/4858<br>anti-PARP (Cell Signaling, 9542S)<br>https://www.cellsignal.cn/products/primary-antibodies/parp-antibody/9542<br>anti-Caspase-3 (Cell Signaling, 9662S)<br>https://www.cellsignal.cn/products/primary-antibodies/caspase-3-antibody/9662<br>anti-NCOA4 (Cell Signaling, #66849)<br>https://www.cellsignal.cn/products/primary-antibodies/ncoa4-e8h8z-rabbit-mab/66849<br>anti-DPYD (Cell Signaling, #4654)

https://www.cellsignal.cn/products/primary-antibodies/dpyd-d35a8-rabbit-mab/4654
anti-DLAT (Cell Signaling, #12362)
https://www.cellsignal.cn/products/primary-antibodies/dlat-4a4-b6-c10-mouse-mab/12362
anti-DLST (Cell Signaling, # 5556)
https://www.cellsignal.cn/products/primary-antibodies/dlst-antibody/5556
anti-NDUFS1 (Abcam, ab169540)
https://www.abcam.cn/products/primary-antibodies/ndufs1-antibody-epr11521b-ab169540.html
anti-NDUFB8 (Abcam, ab192878)
https://www.abcam.cn/products/primary-antibodies/ndufb8-antibody-epr15961-ab192878.html
anti-SDHB (Abcam, ab175225)
https://www.abcam.cn/products/primary-antibodies/sdhb-antibody-epr10880-ab175225.html
anti-UQCRC2 (Abcam, ab203832)
https://www.abcam.cn/products/primary-antibodies/uqcrc2-antibody-epr13051-ab203832.html
anti-MTCO2 (Abcam, ab79393)
https://www.abcam.cn/products/primary-antibodies/mtco2-antibody-epr3314-ab79393.html
anti-ATP5A (Abcam, ab14748)
https://www.abcam.cn/products/primary-antibodies/atp5a-antibody-15h4c4-mitochondrial-marker-ab14748.html
anti-FTH1 (Abcam, ab183781)
https://www.abcam.cn/products/primary-antibodies/ferritin-heavy-chain-antibody-epr18878-ab183781.html
anti-NDUFS3 (Abcam, ab177471)
https://www.abcam.cn/products/primary-antibodies/ndufs3-antibody-epr12782-c-terminal-ab177471.html
anti-UQCRFS1 (Abcam, ab191078)
https://www.abcam.cn/products/primary-antibodies/uqcrfs1risp-antibody-epr16288-ab191078.html
anti-FECH (Abcam, ab137042)
https://www.abcam.cn/products/primary-antibodies/fech-antibody-epr8312-ab137042.html
anti-IREB2 (Abcam, ab181153)
https://www.abcam.cn/products/primary-antibodies/ireb2irp2--aconitase-1aco1-antibody-epr13854-ab181153.html
anti-Tubulin (Proteintech, 10068-1-AP)
https://www.ptgcn.com/products/TUBB3-Antibody-10068-1-AP.htm
anti-ACTB (Proteintech, 81115-1-RR)
https://www.ptgcn.com/products/beta-actin-Antibody-81115-1-RR.htm

Eukaryotic cell lines

Policy information about cell lines and Sex and Gender in Research

Cell line source(s)	The EGFR-mutant human non-small cell lung cancer (NSCLC) cell lines, including H1975, HCC827, PC9, HCC4006, H1650, and A431 were obtained from the American Type Culture Collection (ATCC). All cell lines were cultured in 1640 with 10% fetal bovine serum (FBS, Corning) at 37 °C in a humidified 5% CO2 atmosphere. Before the experiment, the cells were screened for mycoplasma contamination, cross-contamination between species, and authenticity. The cell lines utilized in the experiments were cultured for a maximum of 20 passages.
Authentication	Cell lines were maintained in a centralized cell bank, authenticated by assessment of cell morphology as well as short tandem repeat fingerprinting, and routinely inspected for Mycoplasma contamination using PCR.
Mycoplasma contamination	We confirm that all cell lines were negative for mycoplasma contamination.
Commonly misidentified lines (See ICLAC register)	No commonly misidentified cell lines were used in this study.

Animals and other research organisms

Policy information about studies involving animals; ARRIVE guidelines recommended for reporting animal research, and Sex and Gender in Research

Laboratory animals	Female BALB/c nude mice, 4 weeks old, were purchased from the GemPharmatech. All the animals were maintained in the Nanjing Medical University under specific pathogen-free conditions. All animals were placed under a 12-h light dark cycle. The room temperature was maintained at 22 °C with 55 - 70% humidity. Tumor xenograft models were established in nude mice.
Wild animals	No wild animals were used in this study.
Reporting on sex	To rule out the potential effect of sex on mouse survival, we used female mice in all experiments. In our study, the research involving vertebrate animals and cell lines did not consider gender. The experimental design was based on the lack of reported efficacy, resistance mechanisms, or other gender-related considerations regarding Osimertinib at present.
Field-collected samples	No field-collected samples were used in this study.
Ethics oversight	All studies involving mice were approved by the Animal Care and Use Committee (IACUC) of Nanjing Medical University (IACUC-2012002-1). The maximum tumor size allowed by the IACUC is 20 mm, and none of the experiments exceeded this limit.

Note that full information on the approval of the study protocol must also be provided in the manuscript.

Clinical data

Policy information about clinical studies

All manuscripts should comply with the ICMJE guidelines for publication of clinical research and a completed CONSORT checklist must be included with all submissions.

Clinical trial registration	*Provide the trial registration number from ClinicalTrials.gov or an equivalent agency.*
Study protocol	*Note where the full trial protocol can be accessed OR if not available, explain why.*
Data collection	*Describe the settings and locales of data collection, noting the time periods of recruitment and data collection.*
Outcomes	*Describe how you pre-defined primary and secondary outcome measures and how you assessed these measures.*

Flow Cytometry

Plots

Confirm that:

- [x] The axis labels state the marker and fluorochrome used (e.g. CD4-FITC).
- [x] The axis scales are clearly visible. Include numbers along axes only for bottom left plot of group (a 'group' is an analysis of identical markers).
- [x] All plots are contour plots with outliers or pseudocolor plots.
- [x] A numerical value for number of cells or percentage (with statistics) is provided.

Methodology

Sample preparation

Cell cycle analysis.
H1975, HCC827, PC9, and HCC4006 cells were subjected to Trypsin (Gibco) treatment, as described above, and subsequently harvested. The cells were washed with ice-cold PBS and then fixed overnight at -20°C in 75% ice-cold ethanol before being stained with propidium iodide (PI) for 30 minutes at 37°C. The DNA content of the cells was then measured using a BD Biosciences flow cytometer.
PI/Annexin V apoptosis assay.
Following the procedure outlined in the EdU assay, both floating and adherent H1975 and HCC827 cells were trypsinized and washed with PBS. To detect apoptotic cells, the Annexin V-FITC Apoptosis Detection Kit I (BD Biosciences) was employed, which uses Annexin V-FITC and PI to stain the cells. The manufacturer's instructions were closely followed for the staining process. The cells were then analyzed for the presence of apoptosis using a BD Biosciences flow cytometer.
Cellular ROS analysis.
Following the treatment procedure described above, the cells were incubated with 2 μM CellRox Reagent (Thermo) for 30 minutes at 37°C in the absence of light. The level of ROS in the cells was then quantified using a flow cytometer (BD Biosciences).
For the subcutaneous xenograft model in Figure 6i, a mixture of EGFP-labeled NCOA4 wildtype H1975 cells (2.5 × 106) and mCherry-labeled NCOA4 knockout H1975 cells (2.5 × 106) with 50% Matrigel (Corning, 356234) were subcutaneously injected into the mice. When the xenografts reached approximately 100 mm3, the mice were randomly divided into two groups (five mice in each group) and given Vehicle control or OSI (10 mg/kg, once a day), respectively. The size of subcutaneous tumors was measured every five days, and after 40 days, the mice were sacrificed, and their subcutaneous tumors were extracted and prepared into a single-cell suspension for the analysis of each cell's proportion by fluorescence-activated cell sorting (FACS). To get a suspension of tumor cells, fresh tumors were mechanically and enzymatically disaggregated in dissociation buffer consisting of 1640 medium containing 10% FBS (Corning), 100 U/mL collagenase type IV (Life Technologies), and 50 mg/mL DNase I (Roche). Suspension was incubated at 37 °C for 45 minutes and then further mechanically dissociated. Red blood cells were removed from samples using red blood cell lysis buffer (BioLegend).

Instrument	BD LSRII flow cytometer (BD Biosciences)
Software	Data were collected with BD FACSDiva (version 8.0.1) and analyzed with FlowJo (verson 10.0).
Cell population abundance	At least 20,000 events were counted for each marker.
Gating strategy	FSC SSC to remove debris; SSC-H by SSC-A to define single cells. Positive populations were defined using not stained cells as reference. Isotype controls were used to confirm the specificity of the staining.

- [x] Tick this box to confirm that a figure exemplifying the gating strategy is provided in the Supplementary Information.
